# Effect of vertical load and speed on toothbrush stiffness measurements

**DOI:** 10.1016/j.jfscie.2023.100023

**Published:** 2023-06-14

**Authors:** Ashley N. Bowers, Christina M. Tyrakowski, Jamie Spomer, Prerna Gopal

**Affiliations:** a American Dental Association Science and Research Institute, Chicago, IL; b Now with Perrigo, Allegan, MI

**Keywords:** Toothbrush, bristle stiffness, resistance to deflection, oral health, manual toothbrush, power toothbrush

## Abstract

**Background:**

Dental abrasion can be caused by aggressive brushing or using a toothbrush with stiff bristles. Because this damage to the oral cavity is irreversible, prevention is ideal. The international standards for testing toothbrushes for safety and efficacy are International Organization for Standardization (ISO) 22254, ISO 20126, and ISO 20127. American National Standards (ANSI)/American Dental Association (ADA) 119 is an adoption of ISO 22254 and ISO 20126 for manual toothbrushes, and ANSI/ADA 120 is an adoption of ISO 20127 for powered toothbrushes. ISO 22254 contains a test method for the resistance of the tufted portion to deflection, from which toothbrush stiffness is calculated. Key variables of this test method, such as changing the vertical load (VL) applied to the tufted portion of the brush head during testing and changing the velocity of the brushing table of the toothbrush stiffness test machine, were examined.

**Methods:**

Twenty-eight different manual (n = 5) and powered (n = 23) toothbrushes were tested with modification according to the resistance to deflection method in ANSI/ADA 119. The means of the stiffness values at each VL or brushing table velocity for each toothbrush were compared using 2-sample *t* tests.

**Results:**

Most of the toothbrush stiffness values were statistically significantly different at different VLs. However, most toothbrush stiffness values exhibited no statistical differences between the 2 velocities tested.

**Conclusions:**

Most modern toothbrushes cannot withstand the VL required in the ANSI/ADA standard. The VL used for testing significantly affects stiffness measurements, whereas the velocity of the brushing table during testing did not significantly affect the stiffness values obtained.

## Graphical abstract

**Figure:**
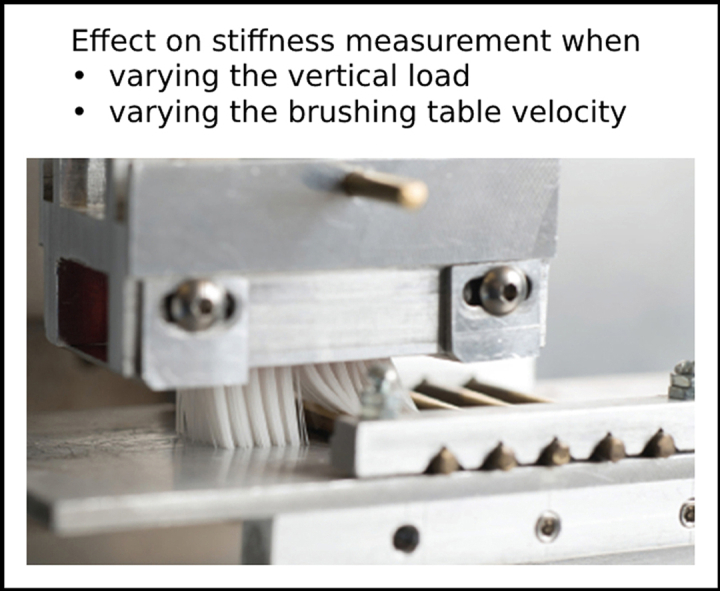

**Why Is This Important?**

With the advancement of toothbrush development and the increasing demand for reliable oral care products, this study examined how toothbrushes are tested for stiffness, part of determining if they are safe for oral tissues. The stiffness of manual and powered toothbrush heads can be calculated by measuring the resistance of the tufted portion to deflection. This novel study aimed to determine how changing the vertical load applied to the toothbrush heads and the velocity of the brushing table affected the resulting stiffness values obtained during testing. This study provides insight into the effect changing those parameters has on the toothbrush stiffness values obtained so that effective updates can be made to toothbrush stiffness test methods that take into account modern manual and powered toothbrush designs.

## Introduction

**Figure 1:**
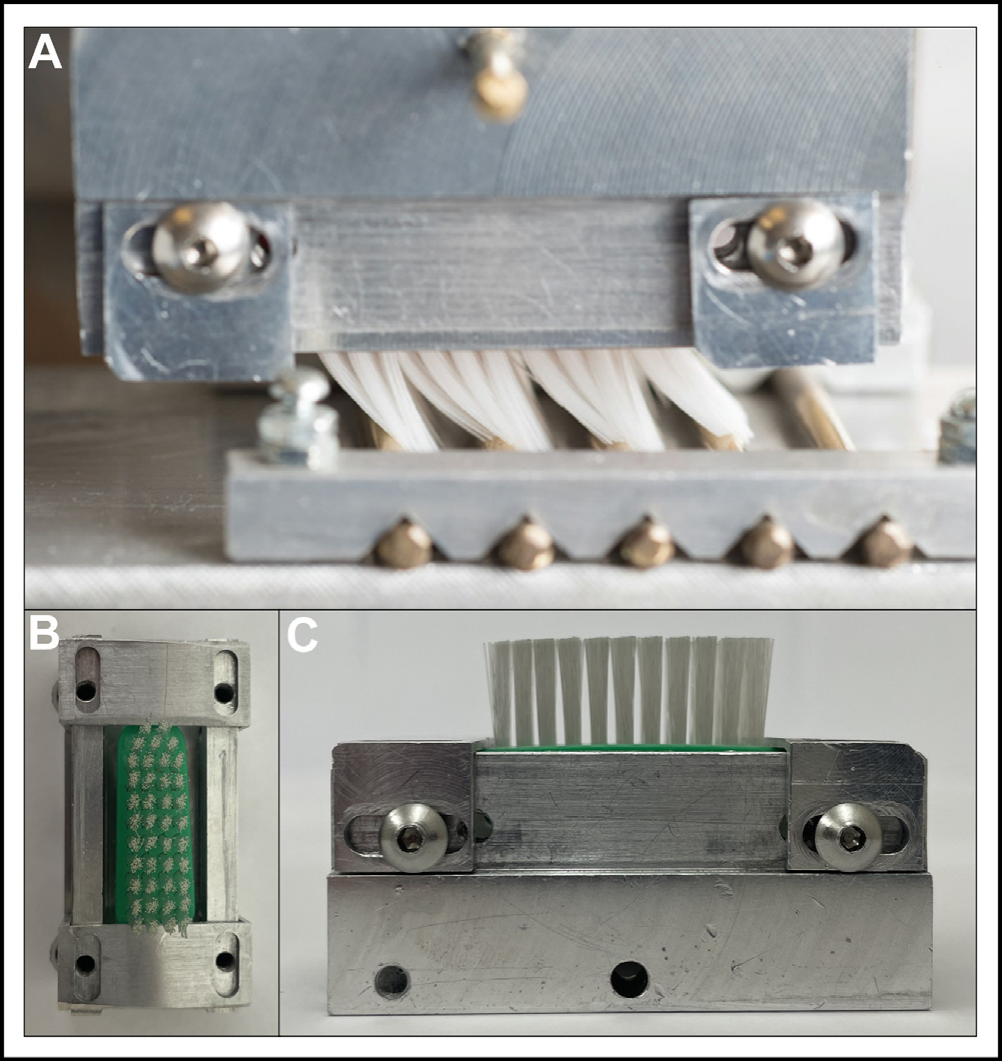
**A.** Manual flat-trim toothbrush in the stiffness testing machine. Not all toothbrushes could withstand higher vertical loads; therefore, some were only tested at lower vertical loads. The bristles moving across the rods yields resistance to deflection measurement. This metal toothbrush holder is for traditional manual flat-trim toothbrushes, as shown in top view (**B**) and side view (**C**). It was used for the testing of toothbrush B.

Toothbrushing is important for maintaining oral health and practicing good oral hygiene. Toothbrush bristle stiffness can potentially contribute to dental abrasion.^1^^,^^2^ Dental abrasion is a type of tooth surface loss or wear, defined as the irreversible loss of the gingival tissues and tooth enamel caused by outside forces applied to the teeth (such as toothbrushing and grinding teeth).^3^^,^^4^ The American Dental Association (ADA) recommends brushing teeth twice daily for 2 minutes with a soft-bristled toothbrush and fluoride toothpaste.^5^ However, one of the predisposing factors of gingival recession is caused by inappropriate toothbrushing.^6^, ^7^, ^8^ Therefore, it is important for people to be aware of how hard they are brushing and of their brushing technique and for manufacturers to evaluate the safety of their toothbrushes before launching products on the market.^9^ The international standards for testing toothbrushes for safety and efficacy are International Organization for Standardization (ISO) 22254, ISO 20126, and ISO 20127.^10^, ^11^, ^12^ American National Standards (ANSI)/American Dental Association (ADA) 119 is an adoption of ISO 22254 and ISO 20126 for manual toothbrushes, and ANSI/ADA 120 is an adoption of ISO 20127 for powered toothbrushes.^13^ Different manual and powered toothbrush styles have been developed with various head shapes, designs, bristle heights, tuft profiles, and sizes.^14^ Modern manual and powered toothbrushes contain an array of bristle arrangements designed differently than the conventional nylon bristle manual flat-trim toothbrush that was first developed in the 1930s.^15^ For this article we are using some standard definitions.^13^ A brush head is the working end of a manual toothbrush to which the filaments are attached, a filament is a single strand within the brush head, and a tuft is a group of filaments gathered together and attached to the brush head.^13^ Furthermore, resistance to deflection refers to the resisting force of the tufts to deflection under a force of 5 N, applied at right angles to the tuft hole plane.^13^ Tuft hole plane refers to the plane between the base of the tufts (where they meet the tufted hole surface) at the top of the brush head and the base of the tufts at the bottom of the brush head (shown in the sFigure [available at the end of this article]).^13^ The tuft hole surface is defined as the surface of the tuft holes, which can be convex, triangular, or plane and limited by a peripheral tangent line to the outer tuft holes.^13^ The tufted area is a linear projection of the tufted surface onto the tuft hole plane or, in other words, the area surrounded by a peripheral tangent line to the outer tufts where they meet the tuft hole plane (shown in the sFigure [available at the end of this article]).^13^ Although there are no international standard definitions for the terms soft, medium, or hard about toothbrush bristles, some manufacturers label their toothbrushes using this terminology.^16^^,^^17^ The American National Standards (ANSI)/ADA and International Organization for Standardization (ISO) standard methods for determining the stiffness of toothbrushes are only applicable to a manual flat-trim toothbrush design, as exemplified in the sFigure (available at the end of this article) and Figure 1.^10^^,^^13^ Resistance to deflection (F), in N, of the tufted area of the toothbrush head (A), in cm^2^, is determined using a test machine, as detailed in the standard, and yields a stiffness value (S_tf_), in newtons per square centimeters according to equation (1).^13^

(1) $$S_{\text{tf}}=\frac{F}{A}$$

Depending on the bristle material and design of the toothbrush head (ie, the diameter of bristles, how many bristles per tuft, and number of tufts per toothbrush head), the resistance to deflection of the tufted portion will vary, which will likewise result in a varying stiffness value in accordance with the formula. For instance, for the measured tufted area of a specific toothbrush, a higher resistance of the tufted portion to deflection during testing will lead to a higher stiffness value. Some of the most important factors influencing toothbrush bristle stiffness are the material the bristles are made from, the bristle length, the bristle diameter, the number of tufts, and bristles per tuft.^14^^,^^18^, ^19^, ^20^, ^21^, ^22^, ^23^ With the advent of modern manual and powered toothbrushes, there is a need to modify the method as new toothbrushes can collapse under the required vertical load (VL) of 5 N and cannot be evaluated for the bristle stiffness according to the method in the standard. These modern toothbrushes were not accounted for in the original version of the standard, but they are still screened using this method in certain instances within the industry because of the lack of alternative recognized test methods. This study examined the effect of incremental changes of the VL applied to the brush head during testing on the stiffness value. This study also analyzed the effect of changing the velocity of the brushing table of the toothbrush stiffness test machine on the stiffness value. As the method was developed for manual flat-trim toothbrushes, these variables and their effect on the stiffness values obtained are being explored to determine if this method can be adapted for modern manual and powered brushes. Five different manual toothbrushes were tested in this study, including a traditional manual flat-trim toothbrush, a large feathered bristle toothbrush, a small flat-trim manual toothbrush, and a small multilevel bristle toothbrush for kids. Twenty-three different powered toothbrushes were also tested with various bristle tuft arrangements, trim profiles such as flat-trim, multilevel and feathered, bristle heights, and brush head sizes and shapes. These toothbrushes cover a wide range of modern manual and powered toothbrushes that consumers use, focusing on smaller manual and powered toothbrushes that tend to collapse under the VL required in ANSI/ADA 119.^13^ This novel study can help inform the standards community and improve the testing methods for these modern toothbrushes. The first part of this study was to determine the effect of the VL on the toothbrush stiffness values obtained, and the second part was to examine the effect the velocity of the brushing table has on the toothbrush stiffness values obtained.

## Methods

### Equipment and toothbrush types

**Figure 2:**
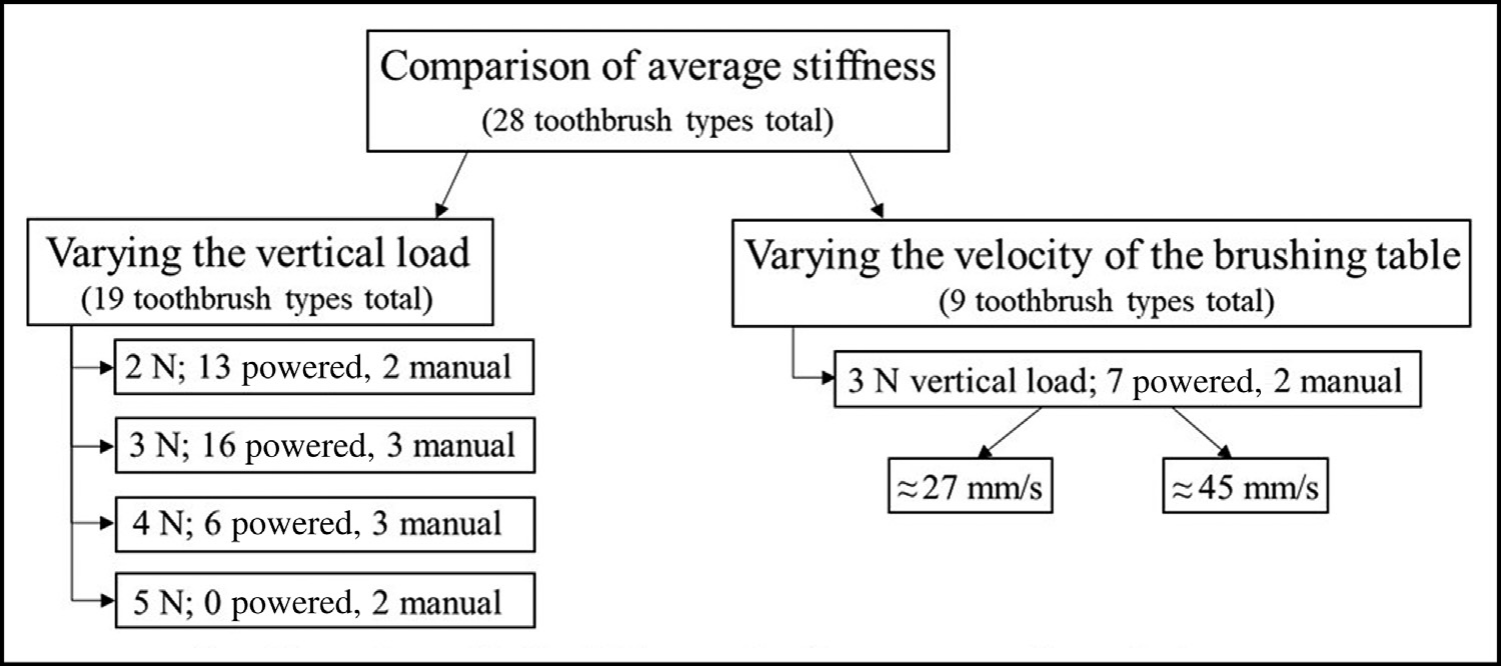
The testing groups from the 28 different toothbrush types tested by varying the vertical load or the velocity. Not all toothbrushes could withstand higher vertical loads; therefore, some were only tested at lower vertical loads.

**Figure 3:**
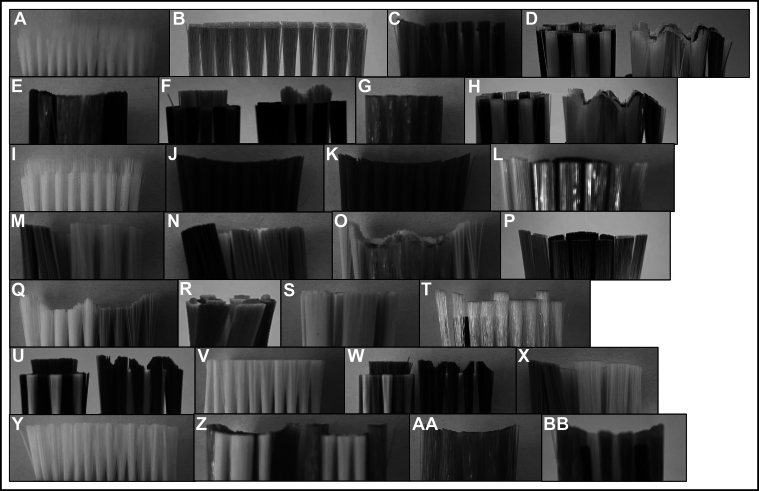
The various types of bristle trim profiles of the manual and powered toothbrushes tested. Letters correspond to the toothbrushes referenced in sTable 1, sTable 2, sTable 3 (available at the end of this article).

**Figure 4:**
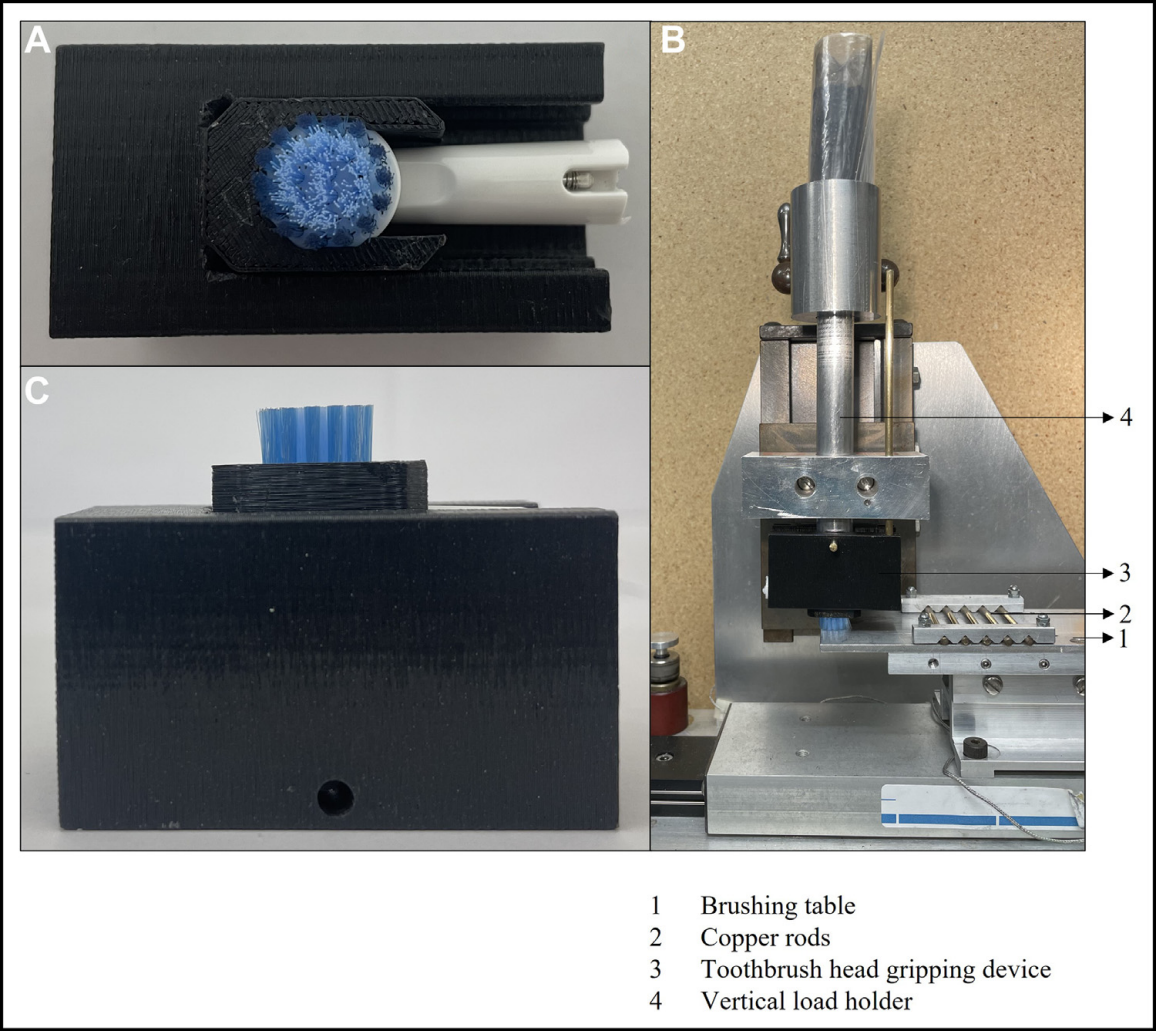
The toothbrush head securely mounted in a 3-dimensional printed (Mojo; Stratasys) (acrylonitrile butadiene styrene [ABSplus-P430; Stratasys]) gripping device that is custom-made for securing the toothbrush head. The gripping device includes an insert that is 3-dimensional printed to fit a specific toothbrush head. Individual inserts for each brush head can be mounted into the base of the gripping device to immobilize the heads, as shown in top view (**A**) and side view (**C**). The gripping device is then attached to the vertical load holder of the stiffness test machine for testing, as pictured in panel **B**. A traditional manual toothbrush in a metal toothbrush holder is displayed in Figure 1.

In total, 28 different manual (n = 5) and powered (n = 23) toothbrushes comprising a variety of toothbrush head sizes, shapes, trim profiles, and bristle heights were obtained and tested. To examine the effects of changing the VL has on the stiffness determination, 19 of the 28 different manual (n = 3) and powered (n = 16) toothbrushes were tested. The other 9 toothbrushes were used in testing the different velocities of the brushing table (Figure 2). Of the 28 toothbrushes, 11 had short bristles, and 17 had long bristles. Four toothbrushes had a flat-trim profile, and 24 toothbrushes had a nonflat-trim profile. Nine toothbrush heads had an overall concave bristle trim profile. Two toothbrushes had tufts that were feathered bristles. Toothbrush sizes ranged from small to large brush heads and from rectangular to oval and round. Figure 3 shows an overview of the types of bristle trim profiles of the manual and powered toothbrushes tested. A traditional nylon bristle manual flat-trim toothbrush was included in the toothbrushes tested during the first study on the effect of changing the VL on the stiffness determination compared with the wide variety of other manual and powered toothbrushes tested. The various manual and powered toothbrushes used in these studies covered a wide range of styles of modern brushes available on the market, such as a small nonflat manual brush for children, a large feather bristle manual brush, a small circular nonflat bristle powered brush, and many other styles of brushes as shown in Figure 3. A toothbrush stiffness test machine (Sabri Dental Enterprises) updated to the ANSI/ADA standard was used. The stiffness machine measured the resistance to deflection, from which the stiffness value was calculated according to ANSI/ADA 119. The stiffness machine has the following components: a brushing table with 5 copper rods, a gripping device that holds the toothbrush head, a holder for the VL to be applied to the toothbrush head, a load cell and a driving unit that moves the table in a longitudinal direction. Several different-sized gripping devices were custom-designed and printed using 3-dimensional printing to ensure a proper fit for the various toothbrush heads (Figure 4).

### Toothbrush conditioning and testing

**Figure 5:**
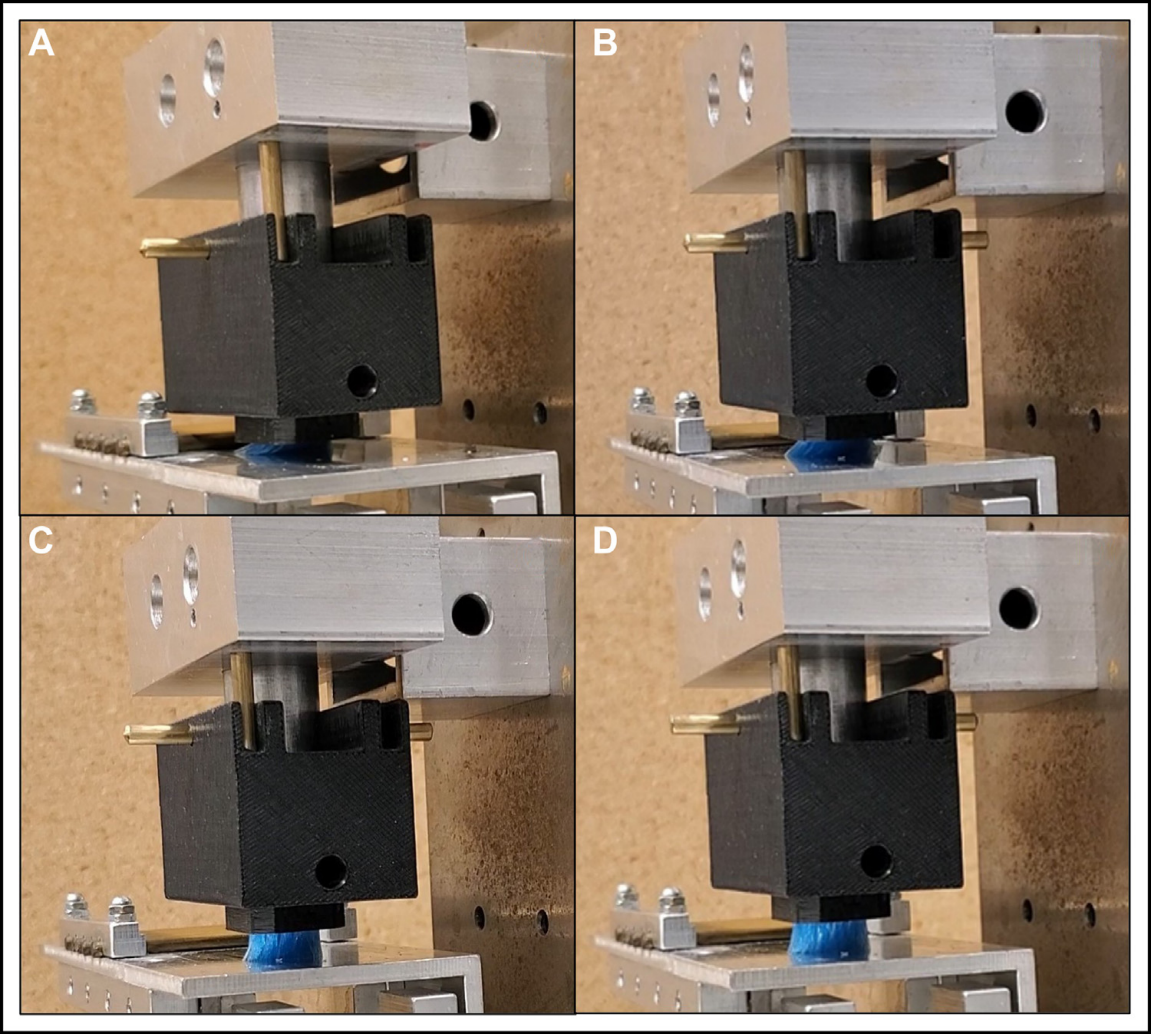
Comparison of toothbrush bristle collapse of toothbrush G under different vertical loads (VLs) (2-5 N). Toothbrush G could only complete testing under 2 N and 3 N VLs; therefore, images were captured after the brush’s first pass over the rods during cycle 1 of the test procedure. **A.** Toothbrush G under 5 N VL. **B.** Toothbrush G under 4 N VL. **C.** Toothbrush G under 3 N VL. **D.** Toothbrush G under 2 N VL.

Before testing, toothbrush heads were cut at the neck with a band saw. Following the sample size outlined in ANSI/ADA 119, 5 samples of the same brush head were adjusted to be approximately the same weight for each test set by sanding the cut neck. Toothbrush heads were then conditioned in water with gentle stimulation (37 °C ± 2 °C, 90 seconds), tapped or flicked to remove excess water, and attached to the test machine. All toothbrushes were tested at room temperature within 3 minutes of removal of the brush head from the water. Brush heads were secured in appropriately sized, custom-made 3-dimensional printed inserts (Mojo; Stratasys) (acrylonitrile butadiene styrene [ABSplus-P430; Stratasys]) that fit into a toothbrush gripping device. Individual inserts for each brush head can be mounted into the base of the gripping device to immobilize the heads (Figure 4). The gripping device is then inserted into the test machine with the VL holder for testing (Figure 4). A traditional manual toothbrush in a metal toothbrush holder is displayed in Figure 1. Toothbrushes were tested according to the resistance to deflection method in ANSI/ADA 119, which is an adoption of the method found in ISO 22254.^10^ To compare the average stiffness value of the tufted area of manual and powered toothbrushes at different VLs, the method was modified with each toothbrush tested at integer VLs ranging from 2 through 5 N (n = 5 for each toothbrush at each VL) for the VLs the toothbrush could withstand. During testing, each toothbrush underwent a reciprocation of sliding back and forth across the rods of the brushing table for 5 cycles at a velocity of approximately 27 mm/s (Figure 1). The force in newtons detected by the load cell was continually captured for the 5 cycles. The resistance to deflection of the tufted portion was measured for each toothbrush at each VL, except when the toothbrush could not support the VL and completely or nearly completely collapsed, from which data was not obtained (as shown in Figure 5). Data were obtained for toothbrushes that only partially collapsed or sagged but could still undergo testing during the 5 cycles. The resistance to deflection of the tufted portion was measured on 5 different toothbrush heads per VL for at least 2 VLs per toothbrush type to observe the effect incremental changes of the VL have on the stiffness value. In a separate study, the effect on stiffness value from changing the speed at which the brush slides across the rods of the brushing table was examined. Testing was performed the same way as previously mentioned, with the exception that the VL was held at 3 N, a VL that all the toothbrushes could withstand, and the speed was tested (n = 5 for each toothbrush at each velocity) at 2 brushing table velocities within the range indicated in the standard (from 10 mm/s-50 mm/s), approximately 27 mm/s and approximately 45 mm/s.^13^

### Calculations and statistical analysis

The stiffness was calculated for each toothbrush using equation (1). The average maximum force in each back-and-forth direction was obtained for each of the 5 brush heads in a set, yielding the resistance to deflection value in newtons. The average resistance to deflection was divided by the tufted area of the toothbrush head, yielding the stiffness value. Analysis was conducted on the effect of incremental changes in force to stiffness value. After testing for normality using Shapiro-Wilk and equality of variances using a 2-sample *F* test, the means of the stiffness values at each VL or each speed for each toothbrush were compared using 2-sample *t* tests with a Bonferroni correction for multiple comparisons. All analysis was conducted in Microsoft Excel, with α set at 0.05.

## Results

### Comparison of average stiffness when varying VL on toothbrushes

Only 2 manual toothbrushes of the 19 different manual and powered toothbrush heads tested could withstand the VL of 5 N required in ANSI/ADA 119, which is applied perpendicular to the tuft hole plane, which contains the toothbrush bristles. Of the 2 manual toothbrushes that could support the 5 N VL, 1 had a traditional flat-trim profile, and 1 was a large toothbrush head with a feathered-trim profile (Figure 3 and sTable 1 [available at the end of this article]). The other manual toothbrush tested in this study, toothbrush C, was for children with a smaller toothbrush head with a multilevel trim profile. Like all the powered toothbrushes in this study, toothbrush C collapsed under the 5 N VL denoted in the standard. This indicates that a modern manual toothbrush showed a similar trend to the powered toothbrushes because of its small size, nonflat trim, or a combination of both. Figure 5 compares the toothbrush bristle collapse of toothbrush G under all VLs (2-5 N). Toothbrush G was a small, circular, flat-trim powered toothbrush. The images were taken after the toothbrush’s first pass over the rods. The full test procedure consists of 5 back-and-forth cycles over the rods, but toothbrush G could not support the 4 N and 5 N VLs during testing and therefore was only tested under 2 N and 3 N VLs (Figure 5). At least 2 different VLs per type of toothbrush were tested. Statistically significant differences were found between stiffness values obtained from comparing 1 VL to the next for most toothbrushes (sTables 1 and 2 [available at the end of this article]). As shown in sTables 1 and 2 (available at the end of this article), of the 26 comparisons, 15 yielded statistically different stiffness values between VLs, with 3 unequal values from manual toothbrushes (sTable 1 [available at the end of this article]) and 12 from powered toothbrushes (sTable 2 [available at the end of this article]). For each toothbrush, there was a general trend in which lowering the VL also lowered the stiffness value obtained.

### Comparison of average stiffness when varying the speed of the brushing table

The ANSI/ADA 119 standard defines the brushing table velocity range from 10 mm/s through 50 mm/s. The second part of this study sought to determine the effect the speed of the brushing table has on the stiffness values obtained while holding the applied VL constant. The first part of this project revealed that only 2 of the 19 toothbrushes tested could withstand the 5 N VL without a complete or near complete collapse of the bristles during testing. Because of this trend, a lower VL (3 N) was chosen to complete the second part of this study. Two different sliding velocities of the brushing table were examined, approximately 27 mm/s, which is around the middle of the standard range, and approximately 45 mm/s, which is at the high end of the standard range, for 9 different manual (n = 2) and powered (n = 7) toothbrush heads. Numerically, it is possible to observe trends in which of the 9 toothbrushes tested, with increased velocity, stiffness decreased for 5 (2 manual, 3 powered) toothbrush heads, stiffness increased for 2 powered toothbrush heads, and stiffness stayed the same for 2 powered toothbrush heads. These differences in the mean stiffness values obtained from both velocities of the brushing table were compared for all 9 toothbrush heads (sTable 3 [available at the end of this article]). Only 1 of the toothbrush heads, a powered toothbrush head, exhibited a statistically significant difference (increase) in stiffness obtained from increasing the velocity of the brushing table (sTable 3 [available at the end of this article]).

## Discussion

### Insight into how the VL affects the toothbrush stiffness determination

Manual and powered toothbrush designs have changed significantly over the years, some of which have smaller, more circular, or different toothbrush head sizes, shapes, and trim profiles than a traditional flat-trim style manual toothbrush.^23^ During toothbrush stiffness testing according to ANSI/ADA 119, the bristles of some modern manual and powered toothbrushes collapse or splay against the copper rods on the brushing table more than others. If bristles are too splayed, sagging, or collapsing against the brushing table and the toothbrush held within the gripping apparatus gets caught on the copper rods of the stiffness machine, then it cannot be tested for an accurate stiffness determination. As a general trend for each toothbrush, lowering the VL also lowered the stiffness value obtained. This was expected because as less force is perpendicularly applied to the tufted plane, the toothbrush bristles have less of a vertical force to resist the horizontal movement on the brushing table during testing. At lighter VLs, some toothbrushes can partially skip across the rods of the brushing table rather than deflect against the rods because of poor contact between the toothbrush and the brushing table. However, there might be an optimal VL, or a small range of ideal VLs, per toothbrush head, based on the individual qualities of the toothbrush head that ensures adequate contact with the rods of the brushing table without either sagging or skipping. The ideal VL would allow the toothbrush bristles to maintain consistent and adequate contact with the brushing table and its rods for the 5 cycles of the test procedure, deflecting over the rods such that the resistance of the toothbrush to deflection can be accurately measured. This study suggests that the VL applied to the toothbrush head during testing is an important parameter of toothbrush stiffness testing. Because the stiffness value obtained was shown to be dependent on the applied force during testing, the accuracy of the stiffness test in ANSI/ADA 119 depends on determining the correct VL for a particular toothbrush head. Arbitrarily lowering the VL when a toothbrush head cannot support 5 N may lead to an inaccurate stiffness determination. Future studies could explore determining the correct VL for a particular toothbrush head when the toothbrush is not of the conventional flat-trim manual design.

### Effect of changing the brushing table velocity on the toothbrush stiffness determination

Another variable in measuring resistance to deflection for toothbrush stiffness determination is the brushing table velocity. Because the ANSI/ADA standard allows a brushing table velocity within a particular range, from 10 mm/s through 50 mm/s, we examined the difference in stiffness values obtained from holding the applied VL at a 3 N force but performing the toothbrush stiffness testing at a brushing table velocity of approximately 27 mm/s and approximately 45 mm/s, covering the middle and high end of the range in the standard. Only 1, a powered toothbrush, of the 9 different manual (2) and powered (7) toothbrush heads tested yielded a statistically significant difference in stiffness values obtained from testing at approximately 27 mm/s vs approximately 45 mm/s. In addition, although not statistically significant, when comparing the stiffness values obtained from testing at approximately 27 mm/s vs approximately 45 mm/s of the 9 toothbrushes, the stiffness values slightly decreased for 5 toothbrushes (3 of which contained nonflat-trim bristles and were powered toothbrush heads and 2 of which contained flat-trim bristles and were manual toothbrush heads of a smaller size than a traditional manual flat-trim toothbrush), whereas it increased for 2 toothbrushes (both of which contained nonflat-trim bristles and were powered toothbrush heads) and stayed the same for 2 toothbrushes (both of which contained a concave bristle profile and were powered toothbrush heads). This suggests that the middle and upper range for the brushing table velocity within the ANSI/ADA 119 standard may be appropriate for accurate stiffness determination of modern toothbrushes within the scope of this study. Future studies could examine the stiffness value obtained from testing in the lower brushing table velocity range of the standard and velocities outside of the range to determine further the effect of brushing table velocity on the obtained stiffness value.

## Conclusions

Toothbrushes have undergone significant design changes to the modern styles popular today. Determining the resistance to deflection of the tufted portion of a toothbrush and the stiffness according to ANSI/ADA 119 remains an important part of evaluating the safety of toothbrushes for consumer use. This study has shown that changing the VL applied to the toothbrush during testing generally yields statistically significant differences between stiffness values when comparing stiffness values obtained from testing at 1 VL to another for each toothbrush. The effect of changing the velocity of the brushing table during testing was another variable examined in this study and showed, for most toothbrushes tested, no statistically significant difference in the stiffness values obtained from testing at 1 velocity compared with another. Future research is necessary to determine if effective updates to the current ANSI/ADA standard method can consider modern toothbrush sizes, shapes, trim profiles, and bristle heights for determining stiffness. Further investigations might also include evaluating the way stiffness is calculated and developing a more clinically relevant stiffness testing apparatus that can account for the various properties of modern toothbrushes.

## Disclosure

None of the authors reported any disclosures.

## Figures and Tables

**sFigure:**
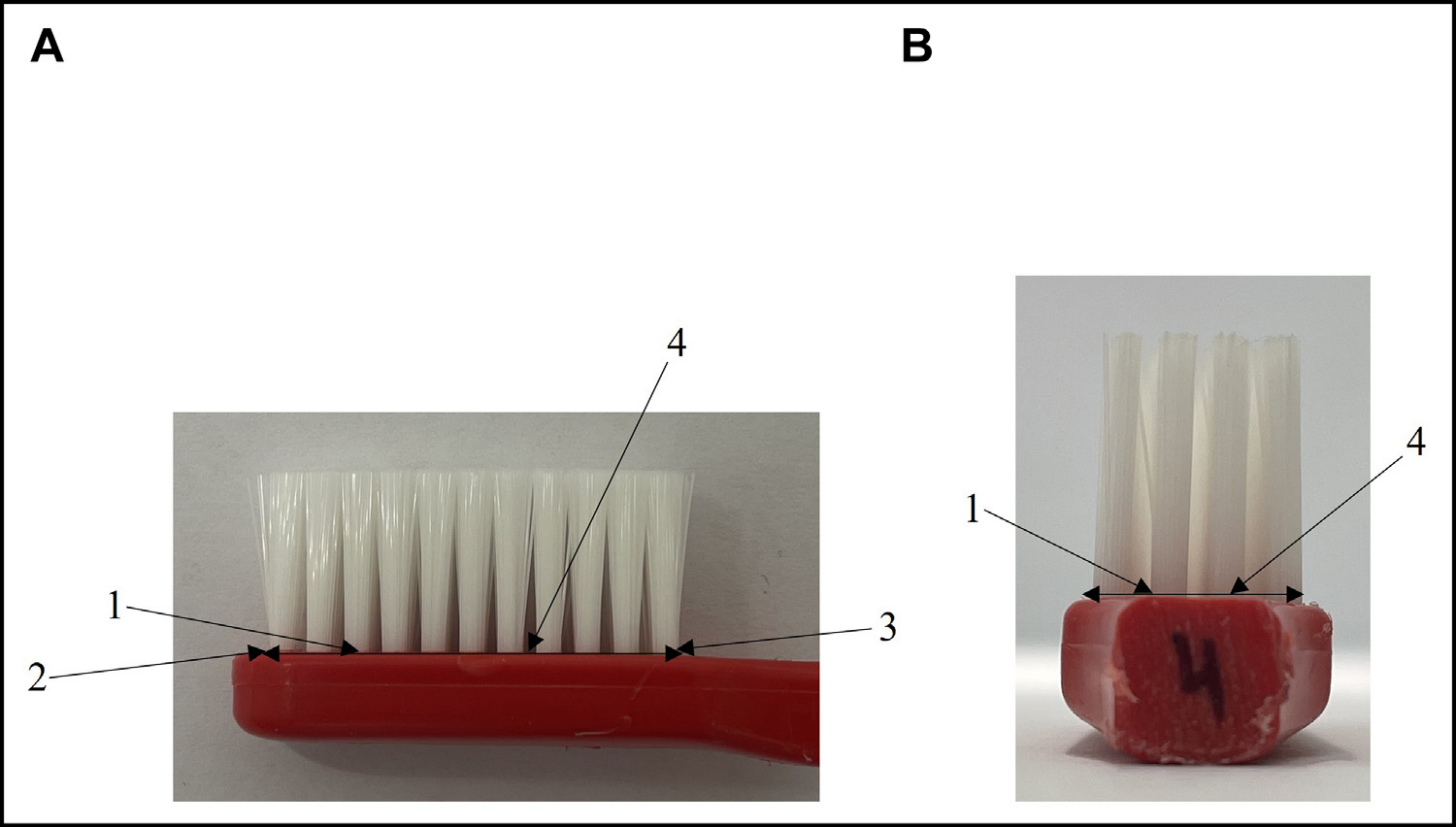
Visual representation of the definitions of a toothbrush. **A.** Side view of the brush head. **B.** Bottom view of the brush head. For this brush, the tuft hole surface (1) is the same as the tuft hole plane (4). (2) is the base of the tufts at the top of the brush head and (3) is the base of the tufts at the bottom of the brush head.

**sTable 1:** Comparison of the average (SD) stiffness of manual toothbrushes using different applied vertical loads.

**Toothbrush**	**Average (SD) stiffness(N/cm**^**2**^**)**		***t*****test*****P*****value(2-tailed)**
**A**	2 N force, 0.70 (0.08)	3 N force, 1.26 (0.08)	< .0001∗
	3 N force, 1.26 (0.08)	4 N force, 1.64 (0.11)	.0002∗
	4 N force, 1.64 (0.11)	5 N force, 1.73 (0.08)	.1759
**B**	2 N force, 1.24 (0.11)	3 N force, 1.34 (0.14)	.2376
	3 N force, 1.34 (0.14)	4 N force, 1.41 (0.15)	.4469
	4 N force, 1.41 (0.15)	5 N force, 1.46 (0.11)	.5543
**C**	3 N force, 1.75 (0.14)	4 N force, 2.56 (0.23)	.0001∗

∗ Stiffness values were statistically different from each other.

**sTable 2:** Comparison of the average (SD) stiffness of powered toothbrushes using different applied vertical loads.

Toothbrush	Average (SD) stiffness (N/cm^2^)		*t* test *P* value (2-tailed)
**D**	2 N force, 2.11 (0.10)	3 N force, 2.48 (0.02)	.0001∗
	3 N force, 2.48 (0.02)	4 N force, 2.83 (0.13)	.0042
**E**	2 N force, 2.91 (0.23)	3 N force, 3.58 (0.05)	.0003∗
	3 N force, 3.58 (0.05)	4 N force, 4.06 (0.25)	.0135
**F**	2 N force, 1.12 (0.14)	3 N force, 1.66 (0.09)	.0001∗
	3 N force, 1.66 (0.09)	4 N force, 2.26 (0.16)	.0003∗
**G**	2 N force, 1.94 (0.15)	3 N force, 2.05 (0.21)	.3632
**H**	3 N force, 2.18 (0.05)	4 N force, 2.61 (0.03)	< .0001∗
**I**	2 N force, 1.60 (0.18)	3 N force, 2.02 (0.08)	.0056
**J**	2 N force, 1.26 (0.07)	3 N force, 1.95 (0.17)	.0004∗
**K**	2 N force, 1.30 (0.04)	3 N force, 1.66 (0.12)	.0016∗
**L**	2 N force, 3.10 (0.29)	3 N force, 3.42 (0.14)	.0649
**M**	2 N force, 2.36 (0.13)	3 N force, 2.89 (0.04)	< .0001∗
**N**	2 N force, 2.21 (0.12)	3 N force, 3.28 (0.14)	< .0001∗
**O**	3 N force, 2.86 (0.17)	4 N force, 3.79 (0.23)	< .0001∗
**P**	3 N force, 2.82 (0.19)	4 N force, 3.18 (0.07)	.0041
**Q**	2 N force, 1.95 (0.29)	3 N force, 2.44 (0.22)	.0183∗
**R**	2 N force, 3.13 (0.11)	3 N force, 4.28 (0.18)	< .0001∗
**S**	2 N force, 2.72 (0.09)	3 N force, 2.95 (0.06)	.0016∗

∗ Stiffness values were statistically different from each other.

**sTable 3:** Comparison of the average (SD) stiffness of toothbrushes with an applied vertical load of 3 N with different velocities of the brushing table.

Toothbrush	Manual or powered toothbrush	Average (SD) stiffness (N/cm^2^)		*t* test *P* value (2-tailed)
		≈27 mm/s velocity	≈45 mm/s velocity	
**T**	Powered	1.49 (0.07)	1.46 (0.06)	.4896
**U**	Powered	1.76 (0.19)	1.45 (0.04)	.1103
**V**	Manual	1.24 (0.12)	1.17 (0.05)	.3910
**W**	Powered	1.63 (0.05)	1.58 (0.04)	.1386
**X**	Powered	2.87 (0.09)	3.46 (0.09)	< .0001∗
**Y**	Manual	1.00 (0.05)	0.92 (0.03)	.0075
**Z**	Powered	3.06 (0.23)	3.30 (0.27)	.1793
**AA**	Powered	2.94 (0.22)	2.94 (0.17)	.9720
**BB**	Powered	2.25 (0.14)	2.25 (0.25)	.9999

∗ Stiffness values were statistically different from each other.
